# Enhanced Xylan/PVA Composite Films via Nano-ZnO Reinforcement for Sustainable Food Packaging

**DOI:** 10.3390/polym17081080

**Published:** 2025-04-16

**Authors:** Lin Yao, Hui Sun, Chang Yu, Yunxuan Weng

**Affiliations:** 1 College of Light Industry Science and Engineering, Beijing Technology and Business University, Beijing 100048, China; 2230402082@st.btbu.edu.cn (L.Y.);; 2 Beijing Key Laboratory of Quality Evaluation Technology for Hygiene and Safety of Plastics, Beijing Technology and Business University, Beijing 100048, China

**Keywords:** hemicellulose, nano-ZnO, sodium hexametaphosphate, barrier properties, biodegradable packaging, sustainable materials

## Abstract

The development of biodegradable alternatives to petroleum-based packaging is essential for environmental sustainability. This study presents a novel approach to enhance the performance of hemicellulose-based films by fabricating xylan/polyvinyl alcohol (PVA) composites reinforced with zinc oxide nanoparticles (nano-ZnO). To address nano-ZnO agglomeration, sodium hexametaphosphate (SHMP) was utilized as a dispersant, while sorbitol improved film flexibility. The composite films were prepared via solution casting, and the effects of nano-ZnO content (0–2.5 wt%) on mechanical, thermal, and barrier properties were systematically evaluated. Results showed that at 2 wt% nano-ZnO loading, the tensile strength increased from 15.0 MPa (control) to 26.15 MPa, representing a 74% enhancement, while oxygen permeability decreased from 1.83 to 0.50 (cm^3^·μm)/(m^2^·d·kPa). Additionally, the thermal stability also improved due to hydrogen bonding and uniform nanoparticle dispersion. At this optimized loading, the hydrophobcity was also maximized, with the contact angle peaking at 74.4° and water vapor permeability decreasing by 18% (1.53·10^−6^·g·h^−1^·m^−1^·Pa^−1^). Excessive nano-ZnO loading (>2 wt%) induced particle agglomeration, generating stress concentrators that disrupted the polymer–nanoparticle interface and compromised mechanical integrity. These findings highlight the potential of nano-ZnO-modified xylan/PVA films as sustainable, high-performance alternatives to conventional packaging. The synergistic use of SHMP and nano-ZnO provides a strategy for designing eco-friendly materials with tunable properties, advancing the use of biomass in food preservation applications.

## 1. Introduction

Petroleum-derived materials have been extensively utilized in packaging due to their cost effectiveness and processability. However, their non-biodegradable nature has led to significant environmental pollution. Consequently, exploring environmentally friendly renewable biomass resources as an alternative to fossil-derived materials, while advancing the research and development of green, biodegradable, eco biomass-based materials has become a central research focus [1,2].

As a widely available biological resource, lignocellulosic materials are extensively used in developing sustainable materials [3,4]. Hemicellulose, the second most abundant component of lignocellulose after cellulose, plays a key role in the preparation of biomass-based materials [5,6]. Its biodegradability, abundance, and broad availability enable application in the pharmaceuticals, food, textiles, and paper industries [7,8,9]. Recent studies highlight its potential application in sensors, green packaging, and bio-based composites [10,11,12].

However, its low degree of polymerization and high hydroxyl group content result in poor mechanical and barrier properties, limiting its application in food packaging. These limitations can be mitigated by utilizing its reactive side-chain groups for physical or chemical modification [13,14]. Physical modification includes adding plasticizers (glycerol and sorbitol) and/or reinforcing agents [15] or blending with polymers [16,17,18] (e.g., polyvinyl alcohol, cellulose, and chitosan), while chemical modifications involve esterification [19,20], etherification, cross-linking [21], grafting [22,23], carboxymethylation [24], and sulfation [17,25,26].

Among reinforcing agents [27,28], nano-SiO_2_, ZnO, and TiO_2_ are of interest in food packaging because of their higher safety and lower cost in comparison with silver nanoparticles [29,30]. Nano-ZnO is particularly promising due to its FDA-approved safety [31]. It also shows advantages including cost effectiveness, high physicochemical activity, and functional properties (including antimicrobial activity, photocatalytic behavior, and photovoltaic properties) [32,33]. However, nano-ZnO’s high surface energy promotes particle agglomeration, compromising its uniform dispersion in hemicellulose matrices and reducing reinforcement efficiency [34]. Sodium hexametaphosphate (SHMP) has been reported to improve nano-ZnO dispersion, enhancing composite performance [35,36].

In this study, PVA/xylan composite films were fabricated via the solution casting method, with nano-ZnO as a reinforcing agent and SHMP as a dispersant. Sorbitol was added to improve flexibility. The effects of nano-ZnO content on mechanical strength, gas/water vapor barrier properties, and hydrophobicity were systematically investigated, demonstrating their potential as sustainable packaging solutions for food products.

## 2. Materials and Research Procedures

### 2.1. Materials

Beech xylan (≥90% purity, M_w_ ≈ 23.5 kDa, hydroxyl content ≈ 30.8 mmol/g), polyvinyl alcohol (PVA, type 1799, M_w_ ≈ 75 kDa, hydrolysis degree ≈ 99%), and D-sorbitol (≥99% purity) were purchased from Shanghai Yuanye Biotechnology Co., Ltd. (Shanghai, China). Zinc oxide nanoparticles (≥99.9% purity, average size ≈ 30 nm, SSA ≈ 35.7 m^2^/g by BET) were supplied by Shandong Keyuan Biochemistry Co., Ltd. (Shandong, China). Sodium hexametaphosphate (analytical grade) was obtained from Fuchen Chemical Reagent Co., Ltd. (Tianjin, China).

### 2.2. Preparation of Nano-ZnO-Modified Xylan/PVA Composite Film

PVA/xylan composite film was prepared via the solution casting method. PVA was dissolved in deionized water (resistivity > 18 MΩ·cm) at 90 °C and stirred for 30 min. Nano-ZnO was ultrasonically dispersed in 0.05% SHMP for 30 min at ambient conditions. Once the PVA blend was cooled to 80 °C, xylan (xylan-to-PVA mass ratio = 2) and sorbitol oil (25% of total solids) were added gradually along with the nano-ZnO dispersion. The mixture was stirred at 80 °C for 3 h, then allowed to settle before cooling to ambient temperature. Subsequently, it was poured into polystyrene plates and air-dried at 25 °C for 24 h. The films were designated as Z0–Z2.5 based on their nano-ZnO content (0–2.5 wt%).

### 2.3. Analytical Methods

#### 2.3.1. FTIR Analysis

FTIR spectra (4000–450 cm^−1^) were acquired using an infrared spectrometer (Waltham, MA, USA) with 16 scans and a resolution of 4 cm^−1^.

#### 2.3.2. SEM Analysis

Samples were cut into small square pieces, sputter-coated with gold, and imaged using a scanning electron microscope (Quanta 250 FEG, FEI, Hillsboro, OR, USA) at an accelerating voltage of 10 kV.

#### 2.3.3. Mechanical Properties Testing

In compliance with Standard GB/T 1040.2–2006 [37], the films were precisely trimmed into rectangular-shaped samples (10 mm × 120 mm) using a cutter, and their thicknesses were measured with a thickness gauge. The mechanical properties were assessed using a digitally controlled universal testing machine (CMT 6104, MTS Systems Co., Ltd., Shenzhen, China), with a 50 mm fixture gap and a tensile rate of 5 mm/min. The average values of tensile properties were recorded, including tensile strength and elongation at break. All measurements were performed in quintuplicate, and results are expressed as mean ± standard deviation.

#### 2.3.4. Thermal Stability Testing

Thermal stability of the films was evaluated by thermogravimetric analysis using an STA 7200 thermogravimetric analyzer (Hitachi, Tokyo, Japan) under nitrogen atmosphere (flow rate: 20 mL/min). The temperature range was set from 40 °C to 650 °C.

#### 2.3.5. Solubility and Swelling Degree

The films were cut into 2 × 2 cm samples and placed in a convection drying oven at 105 °C until a constant weight was achieved (recorded as *W*_1_). The dried sample was immersed in 50 mL of distilled water and maintained at room temperature for 24 ± 1 h. After removal, surface moisture was blotted with filter paper before weighing (*W*_2_). Samples were then re-dried at 105 °C to constant weight (*W*_3_). All measurements were performed in triplicate, with results expressed as mean value.

Solubility and swelling degree were calculated using Equations (1) and (2), respectively. Measurements were recorded with an accuracy of 0.01 g (*W* values in grams) [38].

(1) $$Solubility\;(\%)=\frac{W_{1}-W_{3}}{W_{1}}\times 100\%$$

(2) $$Swelling\;Degree\;(\%)=\frac{W_{2}-W_{1}}{W_{1}}\times 100\%$$

#### 2.3.6. Water Vapor Permeability Testing

The water vapor transmission rate was determined according to GB/T 1037–2021 [39]. A 10 mL glass bottle (effective measurement area: 1.2 cm^2^) was used as the test container. Anhydrous calcium chloride, dried at 105 °C for 2 h and cooled to ambient temperature, was added to the bottle, ensuring a 3 mm gap below the mouth.

A wrinkle-free, intact xylan composite film was sealed over the bottle mouth using a rubber band to maintain 0% internal relative humidity. The bottles were weighed and placed in a desiccator containing saturated distilled water at the bottom. After 24 h, the bottles were reweighed (accurate to 0.0001 g), and a graph of weight gain versus time was plotted, with the slope of the straight line denoted as *K*.

Each group of samples was prepared in triplicate, and the results were expressed as the arithmetic mean of the measurements. The calculation formula was as follows:

(3) $$WVP=\frac{K\times D}{S\times ∆P}$$

where WVP is the water vapor transmission coefficient (10^−6^∙g∙h^−1^∙m^−1^∙Pa^−1^ ); *K* stands for the slope of the resulting straight line (g/h); *D* is the thickness of the composite film (m); *S* is the area of the bottle mouth (m^2^); and Δ*P* is the vapor pressure on both sides of the composite film at 25 °C (Pa) [40].

#### 2.3.7. Oxygen Barrier Properties

According to GB/T 1038–2000, the oxygen transmission rate of films was evaluated using a differential pressure permeability analyzer (VAC-V2, Labthink Instruments Co., Ltd., Jinan, China). Film specimens (10 cm diameter) were tested under 23 °C and 50% relative humidity (RH). The final measurement was derived as the mean of three tests.

#### 2.3.8. Contact Angle

The contact angles were measured using a JY-82B Kruss DSA analyzer (Krüss GmbH, Hamburg, Germany). A 2 μL deionized water droplet was deposited on the film surface, and the angle was calculated via instrument software. Five measurements for each sample were averaged to determine the wettability.

#### 2.3.9. Statistical Analysis

All experimental data are expressed as mean ± standard deviation. One-way analysis of variance (ANOVA) was performed to evaluate the significance of differences between groups, followed by Tukey’s post hoc test for multiple comparisons. Significance levels were set at *p* < 0.05 and *p* < 0.01. Statistical analysis was conducted using SPSS 26.0 (IBM, Armonk, NY, USA).

## 3. Results and Discussion

### 3.1. Fabrication of Hemicellulose-Based Composite Films Incorporating Nano-ZnO

Xylan’s short molecular chain and high glass transition temperature impair film-forming ability, limiting its application alone as a functional film. Blending with other polymers is one of the effective strategies to enhance the film formation characteristics [41]. Polyvinyl alcohol (PVA) is a linear polymer with suitable polarity and affinity due to its rich hydroxyl structure, which is highly compatible with natural polymers for blending [42]. In addition, PVA demonstrates outstanding mechanical strength, resistance to organic solvents, and thermal stability, along with effective gas barrier properties, making it highly beneficial for food packaging applications. Compounding PVA with various biopolymers has been shown to be effective in enhancing material performance and expanding the range of applications.

In the preparation of xylan-based composite films, the addition of PVA significantly enhanced the film-forming ability of xylan and laid the foundation for its potential application in functional food packaging. Meanwhile, sorbitol reduced intermolecular hydrogen bonding, improving flexibility. In addition, to enhance the comprehensive performance of the composite film further, nano-ZnO was incorporated to improve the mechanical and barrier properties. Since nano-ZnO has high surface energy and poor dispersion in the matrix, SHMP addition and sonication ensured uniform nano-ZnO dispersion, thus maximizing reinforcement efficiency [43].

### 3.2. Thermal Stability

**Figure 1:**
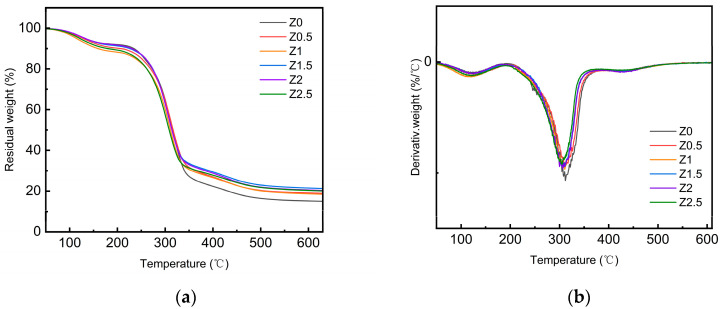
TGA curves (**a**) and DTG curves (**b**) of nano-ZnO-modified xylan/PVA composite films.

The thermal properties of nano-ZnO-modified xylan/PVA films were evaluated by thermogravimetric analysis (TGA) and derivative thermogravimetry (DTG). Preliminary results suggested that ZnO addition improved thermal stability, as indicated by increased degradation temperatures and residue rates. Table 1 and Figure 1 summarize the key thermal decomposition parameters and thermal behavior trends of the samples.

Figure 1 reveals three degradation stages for all films. Pure PVA/xylan composite films show three main stages in the temperature ranges of 30–150 °C, 218–363 °C, and 366–501 °C.

The initial degradation stage (30–150 °C) of ZnO-incorporated composite films was similar to that of pure PVA/xylan films, corresponding to the evaporation of adsorbed water. In the subsequent two stages, the decomposition temperature of nano-ZnO-reinforced films were significantly higher than those of pure PVA/xylan film.

The peak thermal decomposition temperatures (***T***_max2_ and ***T***_max3_) of xylan films increased with nano-ZnO incorporation, indicating enhanced thermal stability. At 70% weight loss, the degradation temperature rose from 338 °C (Z0) to 355–380 °C (Z2.5). This improvement is attributed to hydrogen bonding (between nano-ZnO and the polymer matrix) and nanoparticle-induced heat barriers (retarding volatile release).

### 3.3. Structural and Morphological Analysis

FT-IR analysis was performed to investigate the molecular interactions between nano-ZnO and the polymer matrix. Notable shifts in absorption peaks suggested changes in hydrogen bonding and chemical structure with increasing nano-ZnO content.

**Figure 2:**
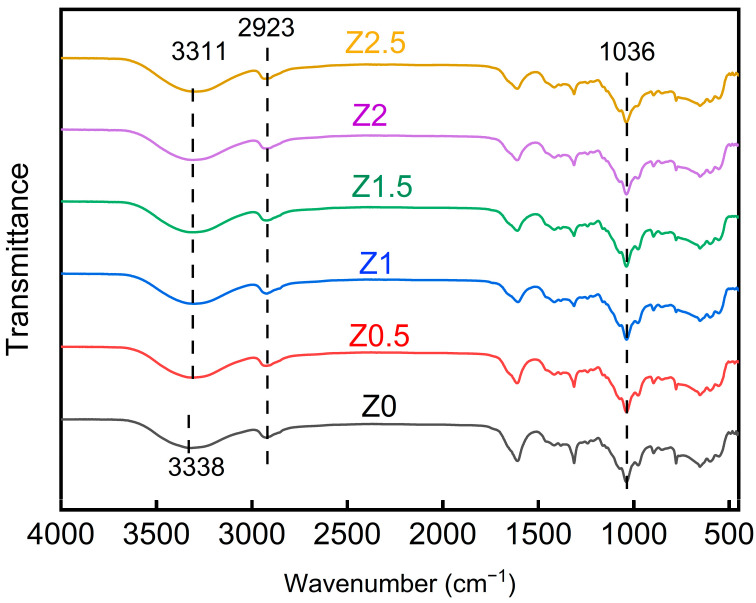
FT-IR spectra of nano-ZnO-modified xylan/PVA composite films.

The FT-IR spectra of nano-ZnO-modified xylan/PVA composite films (Figure 2) revealed characteristic absorption peaks at 3311 cm^−1^ and 3338 cm^−1^ (assigned to O–H stretching vibrations), 2923 cm^−1^ (alkane C–H stretching), 1606 cm^−1^ (residual acetyl groups in xylan), and 1036 cm^−1^ (C–O stretching). Critically, the redshift of the O-H stretching peak from 3338 cm^−1^ to 3311 cm^−1^ provided direct evidence for hydrogen bond formation between nano-ZnO and the polymer matrix.

### 3.4. Surface Morphology Analysis

**Figure 3:**
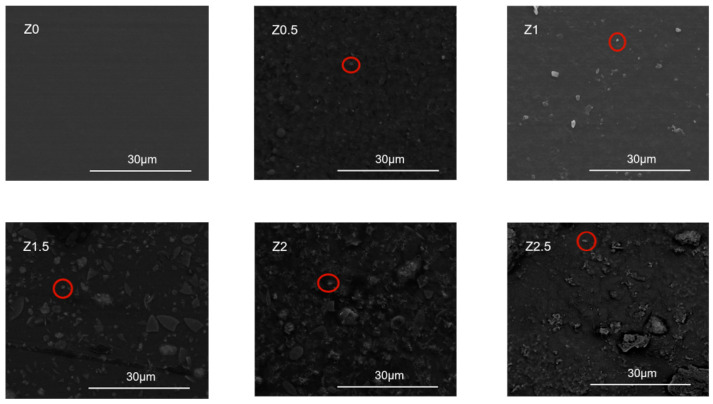
SEM images of nano-ZnO-modified xylan/PVA composite films.

The surface morphologies of ZnO-reinforced xylan/PVA films at different nanoparticle concentrations are shown in Figure 3.

Figure 3 shows the distributions of ZnO nanoparticles in the matrix at different concentrations. The films without added ZnO nanoparticles were smoother, demonstrating that PVA was compatible with xylan. The ZnO nanoparticles were more uniformly distributed when the added concentration was below 2 wt%. With increasing nanoparticle content, granular protrusion gradually appeared on the surface. At higher ZnO nanoparticle concentrations, the granularity on the composite film surface became more pronounced, accompanied by aggregation due to enhanced interparticle attraction. When the addition reached 2.5 wt%, nano-ZnO agglomeration on the surface became more significant, forming larger and more irregular particle agglomerates. This increased surface roughness and consequently reduced mechanical strength. SEM images revealed smooth surfaces for Z0–Z2 but evident agglomeration at 2.5 wt% nano-ZnO (Z2.5, Figure 3), consistent with the observed decrease in mechanical performance.

### 3.5. Mechanical Properties

**Figure 4:**
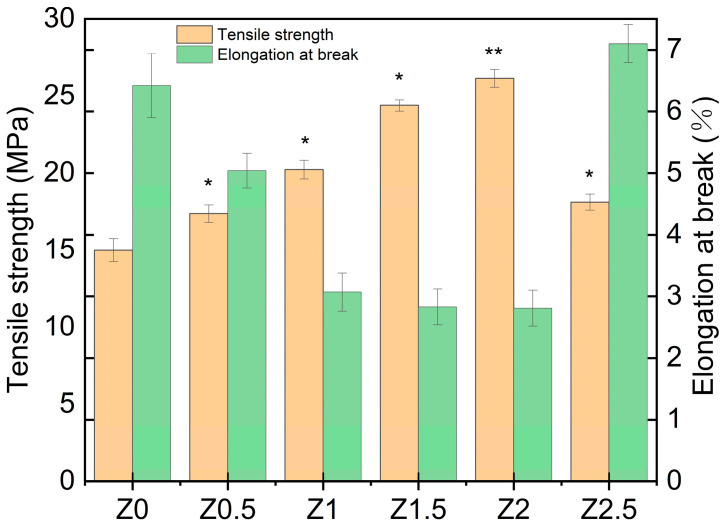
Tensile strength and elongation at break data of nano-ZnO-modified xylan/PVA composite films. Error bars represent standard deviation (*n* = 5). * and ** indicate statistically significant differences from Z0 at *p* < 0.05 and *p* < 0.01, respectively (ANOVA, Tukey’s test).

The incorporation of nano-ZnO notably influenced the mechanical properties of the xylan/PVA composite films. Moderate nanoparticle loading led to a marked enhancement in tensile strength, while excessive addition caused performance decline. These trends are summarized in Figure 4.

As shown in Figure 4, the tensile strength of Z2 (2 wt% nano-ZnO) significantly increased to 26.15 ± 0.58 MPa (mean ± SD), representing a 74% enhancement compared to Z0 (15.0 ± 0.75 MPa, *p* < 0.01). However, when the nano-ZnO content exceeded 2 wt% (Z2.5), the tensile strength significantly decreased to 18.11 ± 0.52 MPa (*p* < 0.05). ANOVA with Tukey’s test results confirmed that Z2 exhibited statistically superior performance over other groups (*p* < 0.05), validating 2 wt% as the optimal loading. When the addition of nano-ZnO was less than 2%, the film was well dispersed owing to the low nano-ZnO content. The uniform distribution of nano-ZnO particles, combined with their high surface energy and large specific surface area, facilitated a significant interfacial region within the composite matrix, thereby strengthening the interaction between nanoparticles and the polymer framework.

The mechanical decline observed at nano-ZnO loadings exceeding 2 wt% (e.g., tensile strength reduction from 26.15 MPa to 18.11 MPa) is attributed to physical agglomeration effects rather than chemical incompatibility. While IR spectroscopy confirms stable hydrogen bonding interactions (O–H stretching at 3311 cm^−1^) and no chemical modification (residual acetyl groups at 1606 cm^−1^), the SEM analysis (Figure 3) and mechanical data conclusively demonstrate that nanoparticle agglomeration creates stress concentrators, disrupting the polymer–nanoparticle interface. This decoupling of chemical stability and physical dispersion explains the apparent contradiction between stable IR profiles and compromised mechanical performance at higher loadings. Further analysis reveals that such agglomeration-induced stress concentrators significantly alter the composite’s molecular interactions. The enlarged nano-ZnO aggregates reduce hydrogen bonding efficiency between nanoparticles and the polymer matrix, while simultaneously expanding the spacing between xylan and PVA molecular chains. This structural slippage diminishes interfacial adhesion, ultimately leading to reduced tensile strength and increased elongation at break, as observed in sample Z2.5.

### 3.6. Oxygen Permeability

In packaging applications, oxygen permeability accelerates food quality deterioration and reduces shelf life. Thus, an effective oxygen barrier is essential for food packaging materials. Table 2 presents the oxygen permeability coefficients of xylan/PVA composite films with varying nano-ZnO content at 23 °C and 50% relative humidity.

Statistical analysis of oxygen permeability (Table 2) revealed that Z2 (0.50 ± 0.06 (cm^3^·μm)/(m^2^·d·kPa)) showed a *p* < 0.05 reduction compared to Z0 (1.83 ± 0.09), confirming the significant enhancement in barrier properties at 2 wt% nano-ZnO. However, Z2.5 exhibited a partial rebound (0.75 ± 0.07), indicating that excessive loading compromises performance (*p* < 0.05 vs. Z2). The oxygen permeability reached a minimum at 2% nano-ZnO [0.5 (cm^3^·μm)/(m^2^·d·kPa)], attributed to reduced free volume and improved film densification. Nano-ZnO forms hydrogen bonds with the PVA/xylan-based matrix, and its nanoscale dimensions enable effective void filling in the composite film, further reducing free volume and enhancing film densification. However, excessive nano-ZnO (>2 wt%) led to slightly reduced oxygen barrier properties due to particle agglomeration.

The oxygen permeability of our composite film [0.5 cm^3^·μm/(m^2^·d·kPa)] is approximately 43% lower than that of birch xylan/CMC films (0.88) reported by Saadatmand et al. [44], and also exhibits better barrier performance in comparison with PLA-based multilayer films reported by Svagan et al. [45]. This enhancement is attributed to the uniform dispersion of nano-ZnO particles and the dense structure observed via SEM (Figure 3).

### 3.7. Water Vapor Permeability

The incorporation of nano-ZnO had a measurable effect on the water vapor barrier properties of the composite films. A downward trend in water vapor permeability was observed with increasing ZnO content, with a minimum value at 2 wt%. Table 3 presents the WVP values for all formulations.

The water vapor permeability (WVP) of Z2 (1.53 ± 0.17 × 10^−6^ g·h^−1^·m^−1^·Pa^−1^) was significantly lower than that of Z0 (1.88 ± 0.05, *p* < 0.05), as determined by ANOVA. No significant differences were observed among Z1, Z1.5, and Z2 (*p* > 0.05). Table 3 demonstrates that nano-ZnO incorporation effectively reduced the water vapor permeability (WVP) of the composite films. This improvement is attributed to the combined effects of water-impermeable nano-ZnO particles and the resulting formation of tortuous water vapor diffusion pathways. The nanoparticles formed additional hydrogen bonds with the PVA/xylan matrix, displacing water molecules at binding sites and creating a more compact structure that restricted water adsorption. The optimal formulation containing 2% nano-ZnO achieved the lowest WVP value of 1.53 × 10^−6^ g·m^−1^·h^−1^·Pa^−1^, representing a significant enhancement in moisture barrier properties.

### 3.8. Surface Wettability

**Figure 5:**
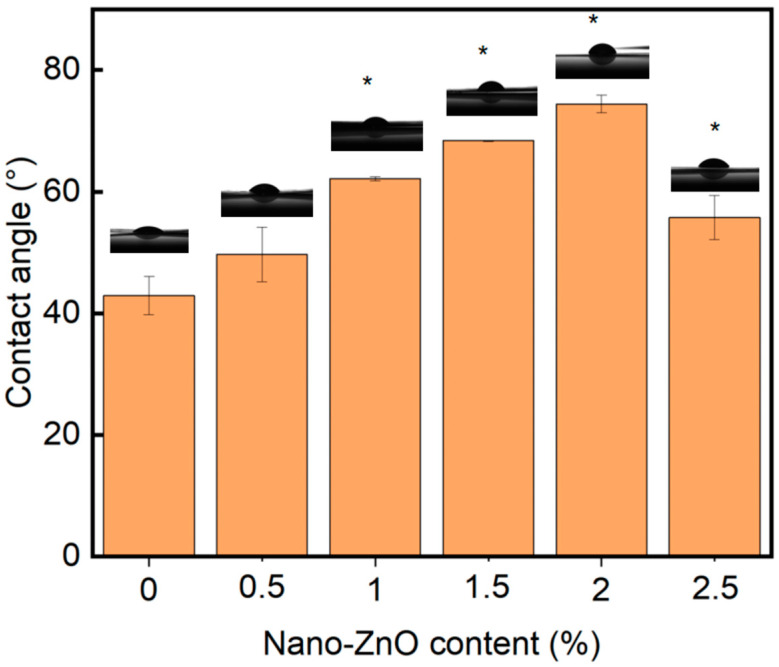
Static contact angles of nano-ZnO-modified xylan/PVA composite films. Error bars represent standard deviation (*n* = 5). * indicates a statistically significant difference from Z0 at *p* < 0.05 (ANOVA, Tukey’s test).

The effect of nano-ZnO on surface hydrophobicity is shown in Figure 5.

The static contact angles were 74.4° (Z2), 68.4° (Z1.5), 62.15° (Z1), 55.75° (Z2.5), 49.7° (Z0.5), and 42.9° (Z0), demonstrating a clear hydrophobicity trend. This improvement stems from two synergistic effects: (1) nano-ZnO particles migrating to the film surface increase roughness, creating air pockets that reduce solid–liquid contact, and (2) decreased surface tension at the film–air interface inhibits water penetration. However, beyond the 2 wt% optimal loading, contact angles declined (e.g., 55.75° for Z2.5) due to nanoparticle agglomeration creating surface inhomogeneity, consistent with the SEM observations in Figure 3.

### 3.9. Swelling Degree and Solubility

Nano-ZnO addition affected both solubility and swelling behavior, with minimum values observed at 2 wt%. Table 4 summarizes these results.

The swelling degree serves as a key indicator of cross-linking density, where higher cross-linking corresponds to lower swelling. As the nano-ZnO content increased, enhanced physical cross-linking formed a denser network structure. The film with 2 wt% ZnO exhibited the lowest swelling degree (156.25%) and solubility (47.43%). This reduction is attributed to increased hydrogen bonding between nano-ZnO and the PVA/xylan matrix, creating tighter intermolecular connections that restrict swelling. However, when the nano-ZnO content exceeds 2%, nanoparticle aggregation within the composite film induces structural discontinuities between xylan and PVA matrices. As a result, the weakened molecular interactions enhanced both its solubility and swelling properties.

## 4. Conclusions

Compared with other biomass-based films such as chitosan/starch composites [46] (~21 MPa), the Z2 film reported in this study exhibited higher tensile strength (26.15 MPa). Additionally, the oxygen barrier performance of Z2 [0.50 (cm^3^·μm)/(m^2^·d·kPa)] was 43% lower than birch xylan/CMC films (0.88) [44]. The increased contact angle (74.4°) also indicates enhanced surface hydrophobicity. SHMP-enabled dispersion was critical for maximizing performance at 2% nano-ZnO loading. The synergistic combination of SHMP-mediated dispersion and nano-ZnO reinforcement establishes xylan/PVA composites as viable alternatives, achieving a balance between mechanical robustness and oxygen barrier capacity. Future research should optimize scalability and assess real-world food preservation efficacy.

## Figures and Tables

**Table 1:** Thermal properties of nano-ZnO-modified xylan/PVA composite films.

Samples	*T*_max_ (°C)			600 °C Carbon Residual Rate (%)
	*T*_max1_ (°C)	*T*_max2_ (°C)	*T*_max3_ (°C)	
Z0	105.35	308.33	420.34	15.20
Z0.5	107.93	309.56	427.02	19.12
Z1	109.32	312.32	423.35	18.65
Z1.5	109.91	309.021	424.45	21.51
Z2	115.72	310.59	430.71	20.55
Z2.5	118.00	309.58	433.78	20.34

**Table 2:** Oxygen permeability results of nano-ZnO-modified xylan/PVA composite films *.

Samples	Oxygen Permeability [(cm^3^·μm)/(m^2^·d·kPa)]
Z0	1.83 ± 0.09
Z0.5	1.00 ± 0.03 *
Z1	0.80 ± 0.07 *
Z1.5	0.67 ± 0.05 *
Z2	0.50 ± 0.06 *
Z2.5	0.75 ± 0.07 *

* Values are expressed as mean ± standard deviation (*n* = 3). Values marked with * indicate statistically significant differences from Z0 at *p* < 0.05 (ANOVA, Tukey’s test).

**Table 3:** Water vapor permeability results of nano-ZnO-modified xylan/PVA composite films *.

Samples	WVP (10^−6^·g·h^−1^∙m^−1^∙Pa^−1^)
Z0	1.88 ± 0.05
Z0.5	1.80 ± 0.20
Z1	1.63 ± 0.14
Z1.5	1.60 ± 0.11
Z2	1.53 ± 0.17 *
Z2.5	1.87 ± 0.13

* Values are expressed as mean ± standard deviation (*n* = 3). * indicates a statistically significant difference from Z0 at *p* < 0.05 (ANOVA, Tukey’s test).

**Table 4:** Swelling degree and solubility data of nano-ZnO-modified xylan/PVA composite films *.

Samples	Solubility (%)	Swelling Degree (%)
Z0	63.94 ± 2.07	219.74 ± 5.98
Z0.5	58.61 ± 4.09	213.52 ± 14.69
Z1	53.89 ± 2.86	211.03 ± 2.77
Z1.5	53.29 ± 5.79	207.46 ± 12.94
Z2	47.43 ± 5.59 *	156.25 ± 10.17 *
Z2.5	53.74 ± 2.58 *	158.90 ± 6.27 *

* Values are expressed as mean ± standard deviation (*n* = 3). Values marked with * are significantly lower than Z0 (*p* < 0.05, ANOVA, Tukey’s test).

## Data Availability

Data are contained within the article.
